# High post‐movement parietal low‐beta power during rhythmic tapping facilitates performance in a stop task

**DOI:** 10.1111/ejn.13328

**Published:** 2016-07-20

**Authors:** Petra Fischer, Huiling Tan, Alek Pogosyan, Peter Brown

**Affiliations:** ^1^ Medical Research Council Brain Network Dynamics UnitUniversity of OxfordOxfordUK; ^2^ Nuffield Department of Clinical NeurosciencesJohn Radcliffe HospitalUniversity of OxfordOxfordOX3 9DUUK

**Keywords:** finger tapping, motor inhibition, sensorimotor synchronization, stop signal

## Abstract

Voluntary movements are followed by a post‐movement electroencephalography (EEG) beta rebound, which increases with practice and confidence in a task. We hypothesized that greater beta modulation reflects less load on cognitive resources and may thus be associated with faster reactions to new stimuli. EEG was recorded in 17 healthy subjects during rhythmically paced index finger tapping. In a STOP condition, participants had to interrupt the upcoming tap in response to an auditory cue, which was timed such that stopping was successful only in ~ 50% of all trials. In a second condition, participants carried on tapping twice after the stop signal (CONTINUE condition). Thus the conditions were distinct in whether abrupt stopping was required as a second task. Modulation of 12–20 Hz power over motor and parietal areas developed with time on each trial and more so in the CONTINUE condition. Reduced modulation in the STOP condition went along with reduced negative mean asynchronies suggesting less confident anticipation of the timing of the next tap. Yet participants were more likely to stop when beta modulation prior to the stop cue was more pronounced. In the STOP condition, expectancy of the stop signal may have increased cognitive load during movement execution given that the task might have to be stopped abruptly. However, within this condition, stopping ability was increased if the preceding tap was followed by a relatively larger beta increase. Significant, albeit weak, correlations confirmed that increased post‐movement beta power was associated with faster reactions to new stimuli, consistent with reduced cognitive load.

## Introduction

Growing evidence associates elevated beta power in motor regions of the cerebral cortex and basal ganglia with reinforcement of the current motor state (Engel & Fries, 2010). Its anti‐kinetic role is particularly apparent in patients with Parkinson's disease (Kühn *et al*., 2006). Conversely, prior to initiating an action, attenuation of beta activity is observed (Williams *et al*., 2005; Van Wijk *et al*., 2009).

Recently it has been reported that decreasing beta after an error may be linked to subsequent behavioural adaptation as it was followed by increased reaction times on the next trial (Jha *et al*., 2015). Simultaneously, after successful stops, a relative beta increase was followed by reduced reaction times as if it reinforced the action that had just been done. In addition, increased post‐movement beta rebound has been shown to be associated with increased confidence in the internal model underlying an action and less reliance on sensory feedback about the consequences of the movement (Tan *et al*., 2014a,b; Tan *et al*. 2016). We set out to investigate the benefits that such reinforcement of actions may bring. If an action is considered adequate for repetition (on the basis of concurrent sensory feedback) and does not need to be changed, then further repetition presumably draws less cognitive resources, which would facilitate faster reactions to novel stimuli. Tapping to a metronome or walking are repetitive actions that require the subject to adapt to incoming sensory information and to continuously monitor both the action and environment. We hypothesized that when these actions do not require adjustment, i.e. when confidence in performing an action at the right time and appropriately is high, beta power between two movements increases more strongly than when the movement is being adapted. Our aim was to determine if beta activity is more likely to be elevated prior to more efficient reactions to new stimuli that dictate a change in the planned motor program, especially when the primary movement is repetitive.

To test this, we selected auditory‐paced rhythmic finger tapping as a primary motor task, and determined how motor performance was affected in anticipation of and in response to a second instruction, which was to abruptly cease tapping. We deliberately selected the pacing interval and timing of the stop signal such that abrupt stopping would be challenging. As the timing of auditory‐paced finger tapping crucially seems to depend on posterior parietal cortex activity (Krause *et al*., 2012, 2014), beta modulation within this region was of particular interest. Furthermore, in Go/NoGo and stopping paradigms, elevated beta in the subthalamic nucleus, inferior frontal cortex and pre‐supplementary motor area has been linked to successful suppression of an upcoming movement (Kühn *et al*., 2004; Swann *et al*., 2009, 2012; Jha *et al*., 2015). We hypothesized that movement‐related beta modulation would grow with the first few taps in each trial as an internal model of the task develops. Conversely, if this modulation indeed reflects the degree of confidence in repeating the same movement, it should be reduced when subjects are preparing themselves to stop the movement abruptly.

## Materials and methods

### Participants

We recorded 21 healthy right‐handed participants after obtaining written consent. Our study complied with Helsinki Declaration guidelines and was approved by the local ethics committee (Medical Sciences Interdivisional Research Ethics Committee, Oxford, MS‐IDREC‐C1‐2015‐090). Right‐handedness was evaluated with the Edinburgh handedness inventory (Oldfield, 1971). Three subjects were excluded because of inaccurate tapping synchronization, as defined as more than 20% of their taps being not within 200 ms of the corresponding pacing sound. One subject had to be excluded because of technical problems with the stop signal delivery time resulting in *n* = 17. The participants analysed were aged between 18 and 38 years (median 26 ± IQR 13 years). Nine participants were female, eight were male.

### Task

We investigated EEG oscillations during the development and abrupt stopping of regular rhythmic tapping with the right index finger, which was guided by an isochronous metronome (700 ms inter‐sound interval, 700 Hz pitch, 40 ms duration).

**Figure 1:**
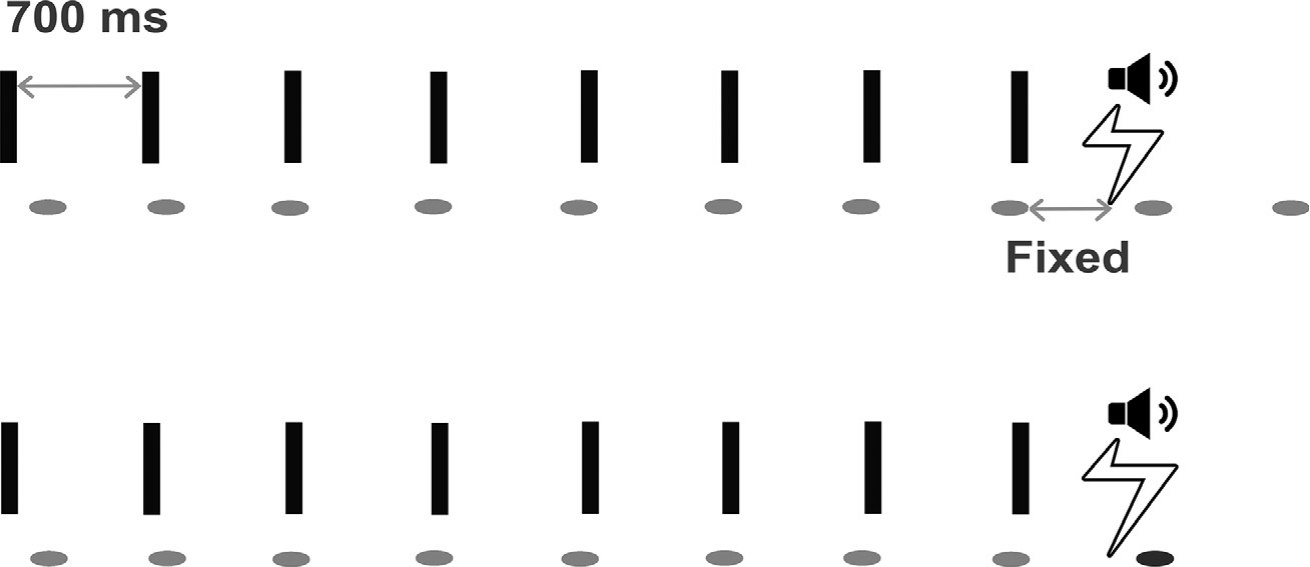
Schematic of the CONTINUE and STOP condition in the upper and lower row respectively. Black rectangles depict metronome sound cues, grey ellipses represent taps. The bolt indicates the stop signal, a sound higher in pitch than the regular tones that was delivered at an individually fixed delay relative to the previous last regular tap. The dark grey rightmost ellipse in the lower panel would represent an unsuccessfully inhibited tap.

Participants were instructed to synchronize their finger tapping to a metronome sound and to start tapping immediately after they heard the first sound. They were asked to make only brief contact with the pressure pad serving as tapping surface and otherwise keep the finger lifted. In addition, they were asked to settle on a movement pattern that was comfortable and to keep the movement amplitude and force the same throughout the whole duration of the experiment. The tapping hand was hidden under an opaque box to avoid visual processing of the movement. Each participant performed the task in two conditions: the STOP condition and the CONTINUE condition (Fig. 1). In the STOP condition, subjects had to resist making one last tap as soon as they heard the stop signal. In the CONTINUE condition, participants were asked to continue with two more taps once the stop signal sounds. The CONTINUE condition was designed to examine the response to the stop signal without sudden movement inhibition, and was therefore without the demand of the same level of cognitive control in terms of alertness and enhanced readiness to stop as in the STOP condition.

The stop signal was a higher pitched tone (2000 Hz pitch, 40 ms duration) delivered after a random number of metronome cues ranging from 6 to 10. Thus, subjects had to stop after 6–10 taps. The minimum number of six sounds was chosen to establish regular cyclic tapping before stopping. Stopping was classified online as successful when the pressure sensor positioned below the finger was not touched.

**Figure 2:**
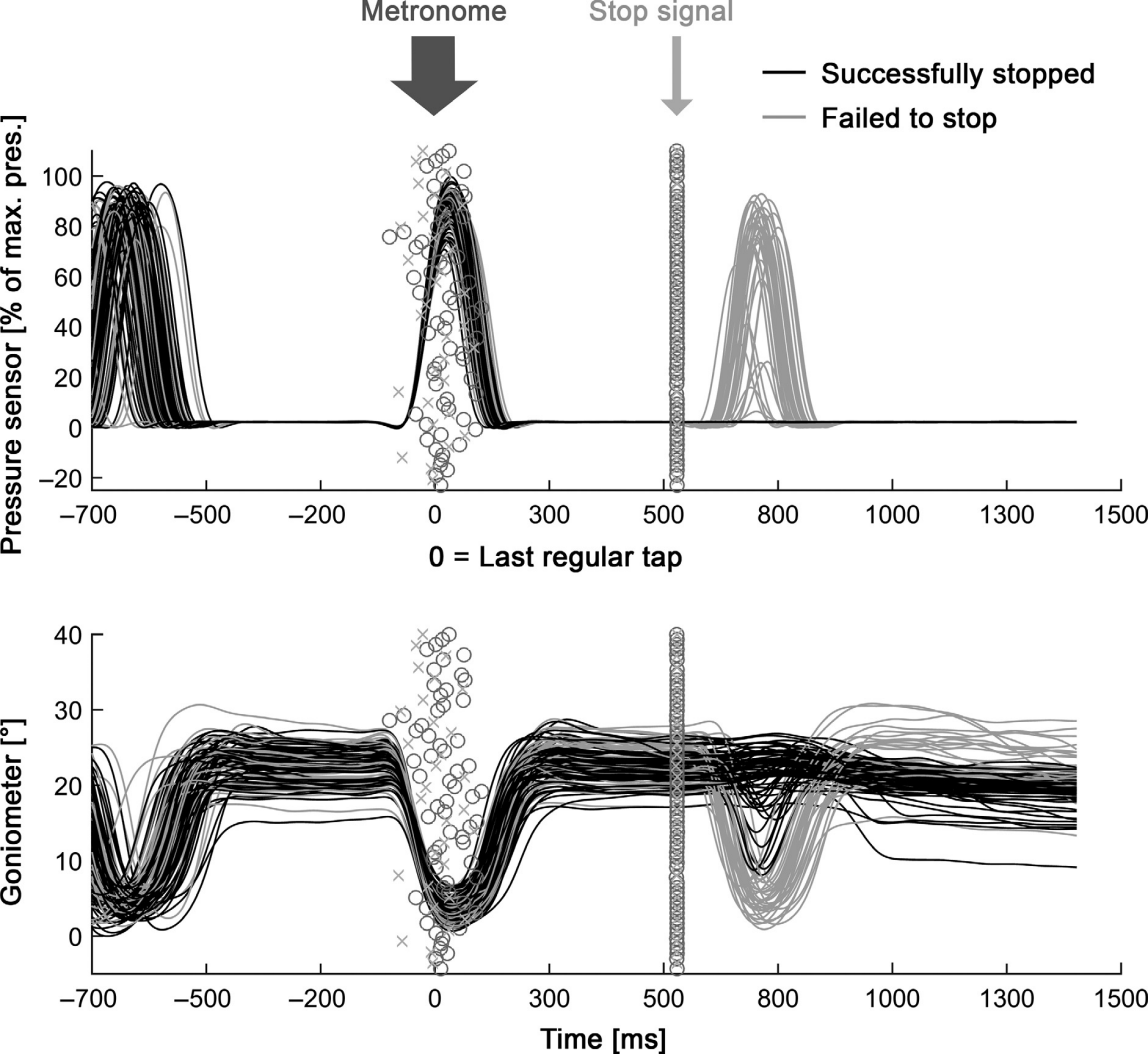
Behavioural data from the first subject. The upper panel shows pressure sensor data. The stop signal was delivered relative to the time of the finger touching the sensor when the pressure signal passed a threshold (time = 0 ms). For this subject, the stop signal delay time was 550 ms relative to 0 ms, marking the last regular tap. Black lines are trials where the tapping movement after the stop signal was successfully interrupted, grey lines are trials where the tap was not inhibited before touching the sensor. The markers around 0 ms represent the temporal offset between the sound and the tap (○ = stopped, × = failed to stop). The markers ○ and × are overlapping rather than separated showing that random fluctuations of the tap‐to‐sound offset were not crucial in determining stopping performance. The lower panel displays the extent of finger flexion as recorded by the goniometer. Note that successfully stopped trials frequently contained downward movement of the finger, which was interrupted timely enough to stop touching of the sensor.

The subject‐specific stop signal delay was determined in an initial training period (20–50 trials) for each participant so that successful interruption of the planned tap would occur in 50–60% of all trials. A success rate that was slightly higher than 50% was favoured to allow for more observations of halfway interrupted taps providing a graded response for subsequent correlative analyses (see Fig. 2). For some participants, performance was more variable, while the time on the task was restricted to about an hour. The actual mean success rate was 58 ± 9% (range = 35–69%). The selected subject‐specific stop signal delay was kept constant throughout the subsequent experimental session of at least 100 trials and ranged between 500 and 565 ms for different participants (544 ± 19 ms). Importantly, in each trial the stop signal was triggered relative to the tap registered by the pressure sensor upon contact (i.e. surpassing a threshold low enough to register each tap), and not to the sound. This was to prevent participants from delaying their taps, which might have otherwise helped them to achieve a better performance. After each block of 10 trials, participants received feedback about the number of successful stops.

Both tasks were practised for at least 10 trials prior to the recording. To help control for slow drifts in performance and EEG measures over time, we recorded the conditions in blocks of 10 trials in the following order:

1. 2 blocks (= 20 trials) CONTINUE, 10 blocks (= 100 trials) STOP, 3 blocks (= 30 trials) CONTINUE

The first CONTINUE block after the STOP blocks was discarded to avoid inclusion of data confounded by conflict, which some subjects might have experienced for a few trials after being trained to stop. Participants were allowed to take breaks in between blocks of 10 consecutive trials whenever they required one. We recorded substantially more trials in the STOP condition to make sure we had enough trials with both successful and unsuccessful stops after discarding irregularly timed taps. In addition, if time allowed, a few more trials were recorded to make up for trials containing artefacts or tapping irregularities. The experiment was programmed in spike 2 (Cambridge Electronic Design) and the auditory cues were delivered via speakers (Creative Inspire T10) at a volume such that the sounds were clearly audible but not perceived as uncomfortable.

### Data acquisition

Data were acquired at a sampling rate of 2048 Hz with a porti tmsi amplifier and porti32 software (data available on request). 23 EEG channels positioned according to the 10–20 system with additional linked earlobes were recorded and referenced to a common average. Electrooculogram (EOG), and three more channels at fronto‐central locations (FCz, FC3, FC4) were additionally recorded. Behavioural data were collected with a goniometer (TMSi Goniometer F35, 1D) attached to the index finger over the metacarpophalangeal joint to measure the degree of finger flexion, and a force sensitive resistor to register the timing upon finger contact with the tapping surface.

### Behavioural data pre‐processing

‘Tap‐to‐sound offset’ was calculated for each individual tap as the difference between the timing of the metronome sound and the timing of tap pressure onset. Taps with an absolute tap‐to‐sound offset larger than 150 ms were excluded to discard taps that were not performed accurately on time with the metronome considering that an offset of 150 ms would exceed three times the average expected negative mean asynchrony in non‐musicians (Krause *et al*., 2010). Furthermore, offsets smaller than 150 ms are shorter than usual reaction times and thus may still be considered anticipatory (Mates *et al*., 1992). Any further behavioural outliers (such as spurious goniometer deflexion) prior to the stop signal were removed following visual inspection. On average 12 ± 10% of all attempted stops and 26 ± 3% of all regular taps were excluded. The goniometer traces and tap‐to‐sound offset distributions were similar and strongly overlapping in successfully stopped vs. unsuccessfully inhibited taps (Fig. 2).

Note that successfully stopped trials frequently contained downward movement of the finger, which was interrupted in time before touching the pressure sensor. This graded performance was captured by a measure we call ‘movement extent’. The measure was computed by normalizing the extent of the downward movement by the amplitude of the upward movement done before. Thus it does not reflect the absolute amount of downward movement but constitutes the relative amount as fraction of the height of the preceding finger lift. 0% movement extent would be a full stop, whereas 50% refers to a movement that was interrupted halfway on the way down. Failure to stop would be captured by a ‘movement extent’ of 100%.

### EEG pre‐processing

Event triggers for tap pressure onset and sound onsets were created in spike 2 (Cambridge Electronic Design). All subsequent analyses were performed in matlab (v. 2014b, The MathWorks Inc., Natick, MA, USA). After drift correction removal (2 s time constant) and manual rejection of artefacts, EEG was re‐referenced to linked earlobes and down‐sampled to 1000 Hz. Eye artefacts were removed by subtracting the filtered and adaptively scaled EOG data (40 Hz low‐pass Butterworth filter with a filter order of 6, passed forwards and backwards). Scaling was performed with an optimization algorithm (matlab function *fminocn*, initial value = 1) minimizing the sum of squared errors. Time‐frequency spectrograms were computed on the continuous data by filtering the data into 3 Hz‐wide frequency bands around centre frequencies shifted by 1 Hz ranging from 3 to 30 Hz [Butterworth, filter order = 6, two‐pass, using the fieldtrip toolbox (Oostenveld *et al*., 2011) functions ft_preproc_lowpassfilter and *ft_preproc_highpassfilter*] and calculating the power of the Hilbert transform.

### EEG processing

#### Evolving reactivity

In each trial, participants performed 6–10 continuous taps. To investigate how power modulation changed over time within a trial, we subdivided taps into early (tap number 2–3), middle (3–4) and late (> 5, excluding taps directly followed by a stop signal) taps within each trial. Behavioural analyses suggested that tapping became more anticipative as the series of taps evolved and that this was more marked in the CONTINUE condition than the STOP condition. Accordingly, we sought progressive changes in the EEG during the tapping series, and differences between these in the two conditions, which might shed light on the neural processes underpinning the behavioural observations.

To assess how modulation evolved in the low‐beta (12–20 Hz) and high‐beta (20–30 Hz) bands, we computed three‐way (2 × 3 × 2) repeated measures anovas on the power modulations. The three factors were task (STOP, CONTINUE), time (early, middle and late taps) and electrode location (C3, Pz). From the STOP condition, 201 early, 204 middle and 201 late taps were included on average per subject given that two taps were collated per trial for the early and middle taps, whereas a variable number (depending on trial length) were collated for late taps. From the CONTINUE condition 84 early, 87 middle and 77 late taps were included.

Modulation of the respective band was quantified by computing first the overall power average in the individual channel (averaged first over all taps for each subject and then over subjects and the two conditions) to determine the time of the maximum and minimum overall power average (including all taps and subjects, averaged across both conditions). These were selected from within a 700 ms time window that started 100 ms before to the tap onset to capture both the minimum around the tap onset as well as the peak of the subsequent increase. Thus, time points denoting maxima and minima (indicated as arrows in Fig. 4) were the same for all time windows in both conditions. In a second step, the difference between the means of activity over 100 ms wide windows centred around these points was computed to obtain the modulation while accounting for small variabilities in peak timings across subjects. The difference was then normalized by the average power within this window.

#### EEG features preceding the stop signal and correlating with performance

To test for correlations in a time window preceding the stop signal, we aligned the EEG data to the onset of the last regular tap registered by the pressure sensor and examined a time window ranging from 150 ms after the tap (disregarding most of the upward movement) to 500 ms. The earliest stop signal delay time within our group was 500 ms, hence the window was restricted to 500 ms to avoid including reactive responses.

### Statistical testing

Pairwise comparisons of the behavioural data for different conditions were performed with paired *t*‐tests or Wilcoxon signed rank tests if the normality assumption was violated. This proved to be the case in a minority of contrasts in Table 1. Normality was assessed with Lilliefors tests. To control for multiple comparisons of several behavioural variables we performed the false discovery rate (FDR) correction procedure, which controls the expected proportion of falsely rejected hypotheses (Benjamini & Hochberg, 1995).

**Table 1:** Differences in the temporal development from the early (2–3) to the late (> 5) taps in the STOP and CONTINUE condition (mean ± SD)

Variable	2–3	4–5	> 5
STOP soundOffset	6.0 ± 26.6	– 46.2 ± 31.0	**– 45.9 ± 32.3**
CONT soundOffset	3.6 ± 32.2	– 52.3 ± 25.9	**− 60.8 ± 28.3**
*P*‐value soundOffset			**(** ***P *** **=** *** *** 0.008*)
STOP downTime	**123.8 ± 42.7**	**121.4 ± 42.8**	**116.6 ± 40.9**
CONT downTime	**141.1 ± 57.0**	**140.8 ± 57.8**	**137.7 ± 54.7**
*P*‐value downTime	**(** ***P *** **=** *** *** **0.002)**	**(** ***P *** **=** *** *** **0.002)**	**(** ***P *** **=** *** *** 0.001*)
STOP ITI_diff	83.9 ± 14.3	9.9 ± 6.5	0.7 ± 4.3
CONT ITI_diff	73.2 ± 21.7	10.3 ± 9.8	**− **1.5 ± 4.7
STOP maxPrs	59.9 ± 14.7	57.4 ± 15.0	58.2 ± 14.4
CONT maxPrs	61.3 ± 14.7	59.0 ± 15.3	58.7 ± 14.7
STOP peakVelDown	159.4 ± 64.6	158.0 ± 63.6	154.8 ± 61.5
CONT peakVelDown	166.4 ± 65.3	163.0 ± 63.8	167.3 ± 65.2
STOP upMvmt	15.0 ± 6.7	14.4 ± 6.5	14.4 ± 6.4
CONT upMvmt	15.7 ± 6.2	15.2 ± 6.1	15.6 ± 5.9
STOP peakVelUp	116.6 ± 65.3	113.2 ± 65.6	114.5 ± 63.6
CONT peakVelUp	114.6 ± 69.3	108.3 ± 66.9	112.5 ± 70.7

Values in bold are significantly different between the STOP and CONTINUE condition after FDR correction. *P*‐values are in brackets below the significant contrasts and were marked with an asterisk if Wilcoxon signed‐rank tests were used instead of *t*‐tests. SoundOffset = offset between sound and tap (negative values represent taps that occurred before the sound), downTime = duration of finger contact with the pressure sensor, ITI_diff = difference between the post‐tap and pre‐tap inter‐tap intervals (ms, positive values denoting longer post‐tap inter‐tap intervals), maxPrs = peak pressure during the tap, peakVelDown = peak velocity of the downward movement of the previous tap, upMvmt = amount of up‐movement, peakVelUp = peak velocity of the upward movement.

Repeated‐measures anovas and post hoc *t*‐tests were performed in spss (v. 22, IBM SPSS Statistics for Windows; IBM Corp., Armonk, NY, USA). If the sphericity assumption was violated, Greenhouse‐Geisser correction was applied and the correction factor ɛ is reported. All other statistical analyses were performed in matlab. Effect sizes reported are calculated based on Cohen's d.

Correlations were calculated as Spearman's rank correlation coefficients. Bootstrapped confidence intervals were computed using the *Spearman* function from the Robust correlation toolbox (Pernet *et al*., 2013). To test in Table 2 whether behavioural correlations were significantly different from zero on a group level, correlation coefficients were Fisher's *z* transformed and underwent a one‐sample *t*‐test (*n* = 17). Partial correlations were computed to control for movement properties when examining the relationship between EEG power and the movement extent. We controlled for each movement variable individually, as well as for two components obtained via principal component analysis (PCA) that explained most of the variance (see Results). Results were considered statistically significant if *P* < 0.05.

**Table 2:** Correlations between movement parameters of the last regular tap and the movement extent after the stop signal (mean±SD)

Variable	Rho ± SD	*P*‐value	Nr. of sub. < 0.05
soundOffset	**0.29 ± 0.13**	**< 0.001**	12
downTime	**− 0.10 ± 0.11**	**0.001**	4
maxPres	**− 0.10 ± 0.09**	**< 0.001**	1
tapNr	**− 0.11 ± 0.16**	**0.012**	5
peakVelDown	0.04 ± 0.12	0.238	3
upMvmt	**0.08 ± 0.11**	**0.013**	3
peakVelUp	**0.07 ± 0.11**	**0.020**	2

Values in bold are FDR‐corrected significant based on one‐sample *t*‐tests of the Fisher's *z*‐transformed intra‐individual correlation coefficients of the 17 subjects. tapNr = number of taps preceding delivery of the stop signal. Remaining variable names are the same as in Table 1.

Before testing for differences in power, each time‐frequency matrix was normalized for each subject by the average across all regular taps (excluding tap 1 and those followed by a stop signal) to obtain a relative power change in percent.

To evaluate statistical significance of mean power differences in time‐frequency spectrograms, we used a cluster‐based permutation procedure: *P*‐values were derived by randomly permuting the assignment of condition labels for the 17 subject averages 1000 times: For each time‐frequency point the *z*‐statistic of the actual mean difference was computed based on the distribution of the 1000 differences resulting from permutation. The resulting *P*‐values were then corrected for multiple comparisons using a cluster‐based permutation approach. To this end, suprathreshold clusters (pre‐cluster threshold: *P *<* *0.05) were determined for each permutation, and the sum of the *z*‐statistics within these clusters was stored to form a distribution of the largest suprathreshold‐cluster values. Finally, the 95th percentile of this distribution served as statistical threshold for the map of the actual *z*‐statistics (Maris & Oostenveld, 2007).

We focussed our analyses on electrodes overlying the contra‐ and ipsilateral motor cortices (C3, C4), pre‐supplementary motor area (FCz) and right prefrontal cortex (F8) because of the role of these regions in motor inhibition (Rae *et al*., 2015) and overlying the parietal cortex (Pz) due to this region's role in anticipatory motor control (Krause *et al*., 2014). Because we selected these regions according to prior knowledge, no additional multiple‐comparison corrections were performed for comparisons across these electrodes.

## Results

### Behavioural results

We hypothesized that tapping would become more regular and anticipative as the series of taps evolved and more so in the CONTINUE condition than the STOP condition, where sudden stopping was required. In addition, we posited that stopping efficiency would also partly depend on how timely or confidently the previous tap had been performed.

First, we determined how tapping characteristics evolved over time on each trial, and whether these characteristics and their temporal evolution differed between the STOP and the CONTINUE condition (Table 1). Taps were split into early (tap number 2–3), middle (3–4) and late taps (> 5, excluding taps directly followed by a stop signal). In both conditions, inter‐tap intervals became more regular over time in each trial as indicated by a reduced difference between two consecutive inter‐tap intervals (ITI_diff).

After the third tap, participants showed a tendency to tap slightly ahead of the sound (soundOffset), which is also known as negative mean asynchrony and can be observed once stable sensorimotor synchronization has developed. Even though tap‐to‐sound offsets were similar in the beginning of each trial in both conditions, the development of the negative mean asynchrony seemed to be more limited when participants were expecting to stop abruptly. This was particularly evident for late taps in the STOP condition when compared to the CONTINUE condition. A 3 × 2 repeated‐measures anova resulted in a significant main effect of time (Greenhouse‐Geisser corrected *F*_2,32_ = 81.0, ɛ = 0.56, *P *<* *0.001) and interaction between condition and time (Greenhouse‐Geisser corrected *F*_2,32_ = 5.2, ɛ = 0.64, *P *=* *0.026) and no significant main effect for condition (*F*_1,16_ = 3.8, ɛ = 1.0, *P *=* *0.070). These findings suggest that the development of anticipatory tapping was relatively compromised when participants expected stopping and particularly, when expectancy of the stop signal was at its height after the 5th tap. In addition to this, the time spent in contact with the pressure sensor was shorter (downTime) in the STOP condition than in the CONTINUE condition straight from the start of each trial. The significant interaction (Greenhouse‐Geisser corrected *F*_2,32_ = 4.8, ɛ = 0.95, *P *=* *0.017) in a 3 × 2 anova indicates that in addition to the main effect of condition (*F*_1,16_ = 15.0, ɛ = 1.0, *P *=* *0.001) and time (Greenhouse‐Geisser corrected *F*_2,32_ = 5.0, ɛ = 0.80, *P *=* *0.020) this contact period was shortened even more in the STOP condition.

**Figure 3:**
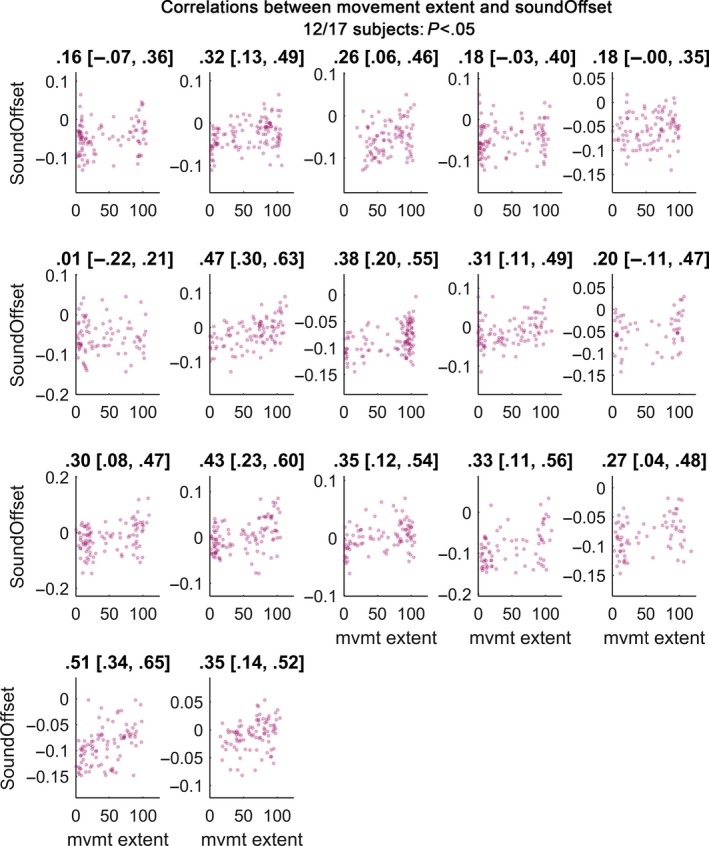
Scatter plot of correlations between movement extent (*x*‐axis) and tap‐to‐sound offset (*y*‐axis). Subplots show individual participants. Plot titles denote Spearman's rho followed by its 95% bootstrapped confidence interval. Participants were less likely to stop when the last regular tap was relatively late with respect to the metronome sound. 12 of 17 subjects had significant correlations.

Second, within the STOP condition we sought evidence to support the hypothesis that stopping efficiency would be partly dependent on the degree of confidence in the internal model underlying the tapping by examining the relationship between the movement parameters of the preceding tap and stopping success, and quantifying this via rank correlation coefficients (Table 2). If participants tapped relatively early in comparison to the metronome sound instead of lagging behind, stopping was more successful (soundOffset, *P *<* *0.001, 12 of 17 subjects significant). This relationship is visible in the scatter plot of individual participants in Fig. 3. If the last tap was relatively vigorous (maxPres, *P *<* *0.001), with the finger in relatively long contact with the pressure sensor (downTime, *P *=* *0.001), then stopping was also more successful. Note though, that a significant relationship with maxPres or downTime was only present in 5 of the 17 subjects in total (Supporting Information Figs S1 and S2). In most participants, the sign of the correlation was negative, hence the significant group level results; but compared to the 12 of 17 significant correlations with soundOffset, the relationship between tapping vigour and movement extent was clearly weaker.

The more taps participants performed prior to presentation of the stop signal, i.e. the later it was delivered in a series, the easier it was for subjects to stop (tapNr, *P *=* *0.012, 5 of 17 subjects individually significant) consistent with growing expectancy of the stop signal. Thus, evidence of increased confidence in terms of less hesitant tapping and increased anticipation of the metronome (in the form of negative asynchrony) were associated with greater stopping efficiency.

Prior to successful stopping, the movement also seemed more controlled considering the smaller upward movement of the tapping finger (upMvmt, *P *=* *0.013, 3 of 17 subjects individually significant), which directly relates to its speed (peakVelUp, *P *=* *0.020, 2 of 17 subjects individually significant).

### Electroencephalography

Power modulation within a tap‐cycle occurred predominantly in theta and high‐beta frequencies (Fig. 5A). After the tap (at 0 ms), power increased first in the theta band. 20–30 Hz beta activity decreased after the tap onset and then peaked right in the middle between two consecutive taps and was most strongly modulated in electrodes overlying the contralateral motor cortex (C3) and preSMA (FCz). High‐beta modulation was less pronounced over parietal cortex (Pz). For subsequent quantitative analyses of spectral changes we therefore selected C3 and Pz as representative of these two patterns of EEG response.

### EEG results: evolving reactivity

#### Low‐beta power

**Figure 4:**
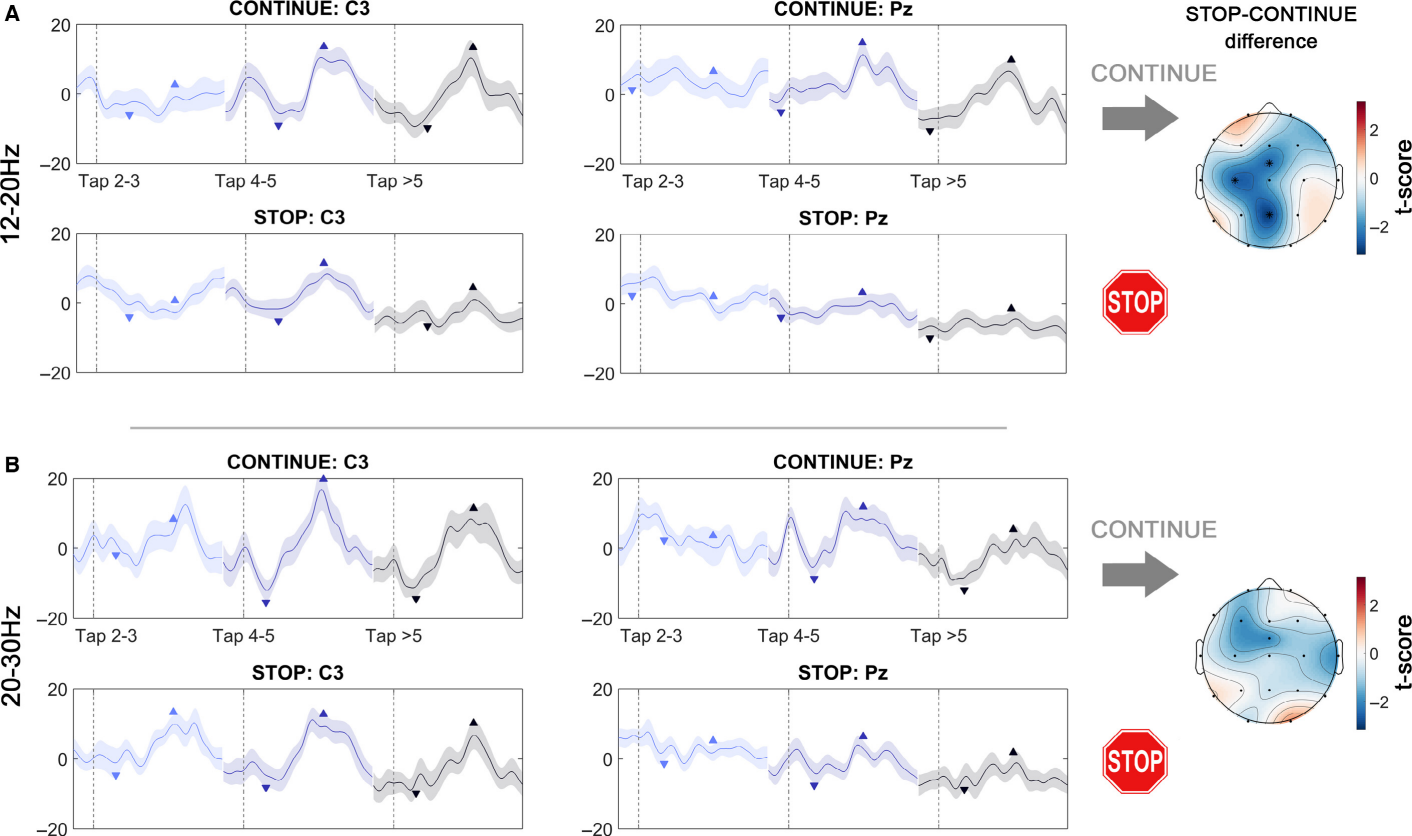
Temporal development of the % change in (A) 12–20 Hz and (B) 20–30 Hz power. The left and right column depict modulation in the electrodes C3 and Pz respectively. Data are aligned to taps as denoted in the legend showing one tap‐cycle within a – 100 : 600 ms window. The left, middle and right traces depict early (2–3), middle (3–4) and late (> 5) taps respectively. Downward arrows denote the location of the average power trough, and upward arrows denote the location of the average power peak, which was used to compute the modulation. In (A) the 12–20 Hz modulation developed in the CONTINUE condition (upper row) after the third tap both in C3 and Pz. In the STOP condition, modulation was strongly attenuated, particularly in Pz. In (B) the average 20–30 Hz modulation was again stronger in C3 than in Pz, and modulation increased after the third tap. Topoplots to the right show the distribution of negative *t*‐scores of the condition differences in modulation, which were significant in Pz, C3 and FCz for the low‐beta band.

The anova with low‐beta power as dependent variable (Fig. 4) resulted in two significant main effects of time (*F*_2,32_ = 14.2, ɛ = 0.95, *P *<* *0.001) and condition (*F*_1,16_ = 10.7, ɛ = 1.0, *P *=* *0.005, mean_CONTINUE_ = 7.8, mean_STOP_ = 1.2): average modulation steeply increased from early taps to taps 4–5 (mean_early_ = − 2.5, mean_middle_ = 8.0, mean_late_ = 8.0; *P*_early vs. middle_ < 0.001, *P*_early vs. late_ = 0.001, *P*_middle vs. late_ = 0.993). Figure 4A shows how the 12–20 Hz modulation developed in the CONTINUE condition after the third tap both in C3 and Pz. In the STOP condition, however, this modulation appeared attenuated in C3 and even more so in Pz, although it should be noted that we did not find a significant main effect of electrode location (*F*_1,16_ = 2.0, ɛ = 1.0, *P *=* *0.173) nor significant interactions (*P*_loc*cond_ = 0.349, *P*_loc*time_ = 0.432, *P*_cond*time_ = 0.507, *P*_loc*cond*time_ = 0.954).

To explore the validity of the comparison between the STOP and CONTINUE condition, we performed two additional control analyses. First, we verified that neither tapping parameters nor low‐beta modulation differed between the first and second block of the CONTINUE condition (see Supporting Information 2.1). Second, we made sure that the condition difference did not result from a different number of trials. To this end, trials from the two conditions were matched in number by selecting a reduced subset of trials from the middle of the STOP condition, discarding the same amount of data at the beginning and end of each participant's recording block (see Supporting Information 2.2).

#### High‐beta power

The anova with 20–30 Hz power as dependent variable (Fig. 4B) resulted in two significant main effects of electrode location (*F*_1,16_ = 8.7, ɛ = 1.0, *P *=* *0.009) and time (*F*_2,32_ = 17.5, ɛ = 0.98, *P *<* *0.001). Power over C3 was more strongly modulated than over Pz (mean_C3_ = 13.1, mean_Pz_ = 4.0), and modulation was again established more strongly after the third tap (mean_early_ = 1.8, mean_middle_ = 13.1, mean_late_ = 10.7; *P*_early vs. middle_ < 0.001, *P*_early vs. late_ < 0.001, *P*_middle vs. late_ = 0.286).

As additional control, to evaluate whether the condition difference is specific to the low‐beta band, we performed the same anova for 8–12 Hz alpha modulation, and found no significant main effects (*P*_channel_ = 0.173, *P*_condition_ = 0.219, *P*_time_ = 0.719) or interactions (*P*_chan*condition_ = 0.337, *P*_channel*time_ = 0.538, *P*_condition*time_ = 0.521, *P*_channel*condition*time_ = 0.450).

In summary, movement‐related modulation in both low‐beta and high‐beta frequency ranges increased in C3 and Pz with the number of taps. We only observed condition differences in the low‐beta band, showing that modulation of low‐beta power was reduced in the STOP condition, where participants had to be prepared to interrupt their movement quickly and where negative mean asynchrony was smaller.

### EEG results: features preceding the stop signal and correlating with performance

Our behavioural results raised the possibility that stopping success was partly dependent on the timing of the last regular tap relative to the metronome and in some also on tapping vigour. Our EEG findings suggested that the gradual increase in low‐beta reactivity might be a candidate marker for the neural processes underpinning the development of stable sensorimotor synchronization, which seemed to depend on how confidently or cautiously the ongoing rhythmic task was performed. If correct, then just as stopping efficiency was partly dependent on how timely and confidently the last tap was performed as shown in behaviour, we would expect power in the low‐beta frequency band to correlate with stopping efficiency.

**Figure 5:**
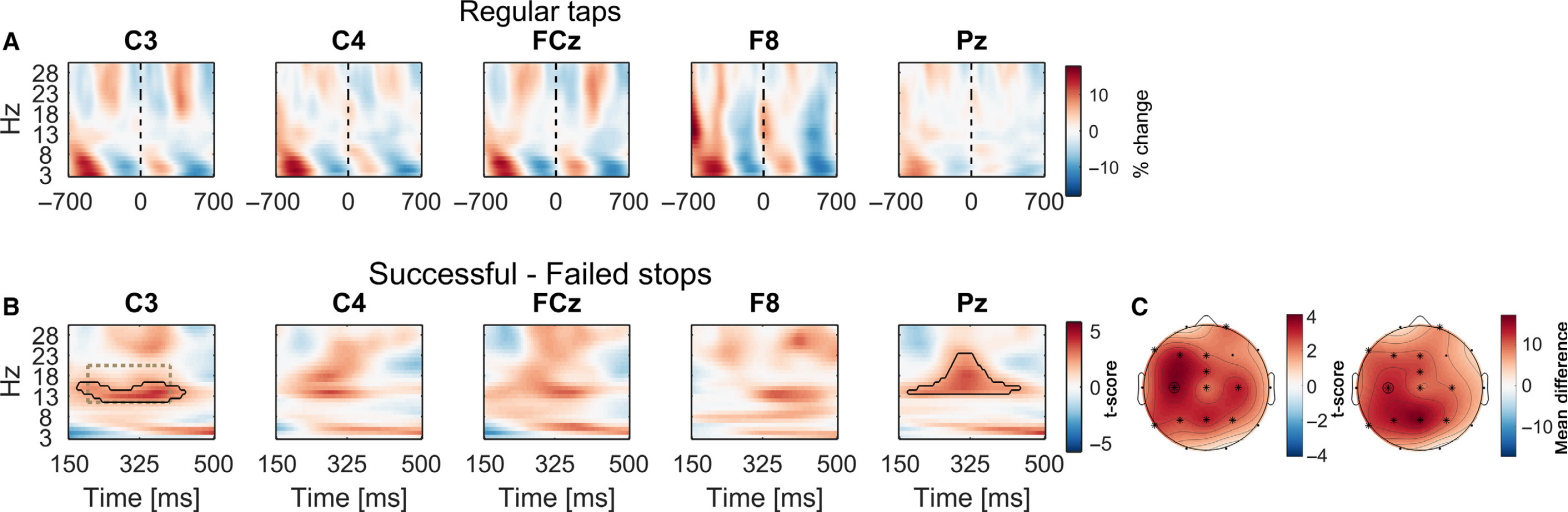
EEG preceding the stop signal. (A) Power aligned to regular taps (time = 0) in the STOP condition. The black dashed line denotes the finger contact with the pressure sensor. Power was *z*‐transformed for each frequency within the time window displayed before being averaged across subjects for better visual display. (B) *t*‐Scores of power differences between successful and failed stops prior to the stop signal. Clusters surrounded by black outlines denote that power was significantly higher when participants interrupted their movement more successfully (movement extent threshold < 40%). (C) Topoplots show the distribution of *t*‐scores and mean differences. In locations marked with a star, 12–20 Hz beta was significantly higher prior to successful stops averaged within the window outlined by the dashed rectangle in (B) in C3. The channel location of C3 in the topoplots is highlighted with a black circle surrounding the star.

**Figure 6:**
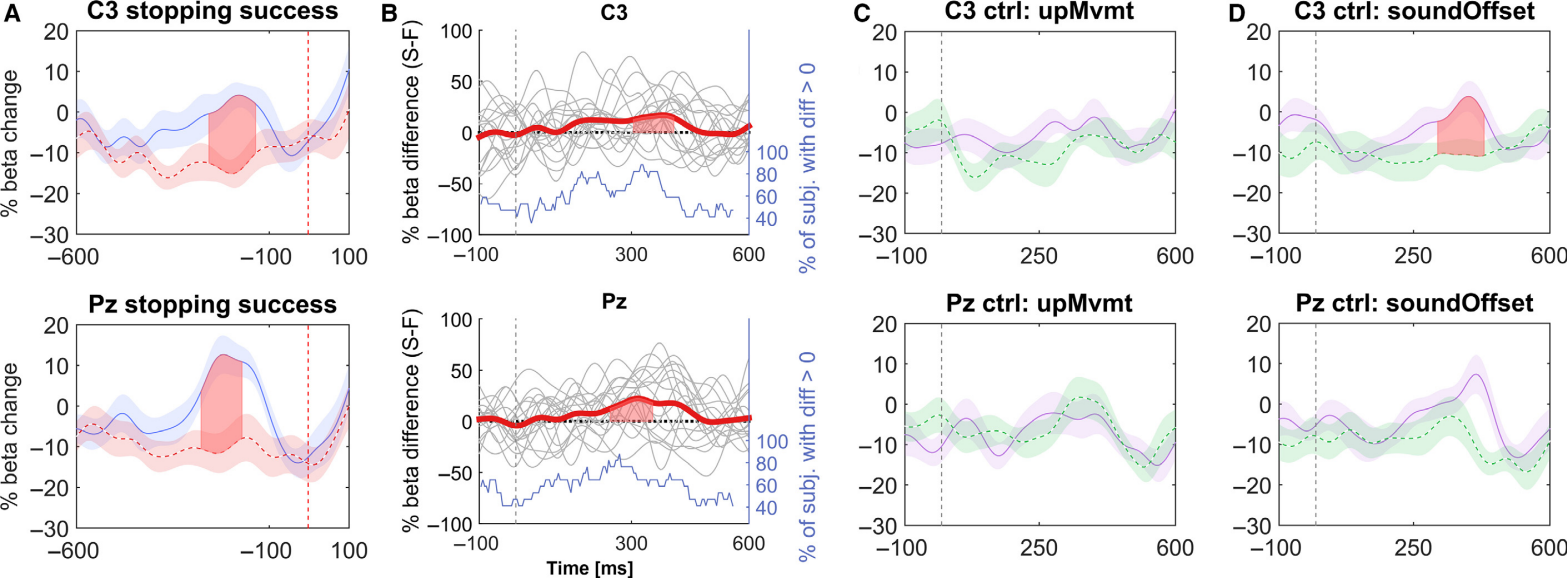
(A) 12–20 Hz beta power time course following the stop signal (vertical dashed line). Data were subdivided according to stopping performance (movement extent threshold < 40%: solid curve; > 40%: dashed curve). (B) Time courses of individual differences between the power of successful and unsuccessful stops subdivided as in (A). Data are aligned to the last regular tap (vertical dashed line) preceding the stop signal. The bold line denotes the mean difference. The bottom stepped line shows for each point in time the fraction of participants who had higher beta power prior to successful stops. (C) Data are aligned to the last regular tap and median split according to the amount of upward movement *upMvmt* (smaller *upMvmt* = solid curve; bigger *upMvmt* = dashed curve). (D) Data are aligned to the last regular tap and median split according to *soundOffset* (more negative, i.e., anticipatory *soundOffset* = solid curve; more positive, i.e., delayed *soundOffset* = dashed curve). Filled areas between lines indicate significant differences, which were cluster‐based multiple comparison corrected. Shaded error bars around curves denote standard errors. Note that stopping the finger taps was associated with a pattern of beta power modulation that was more similar to the CONTINUE condition; compare the solid trace of the successful stop beta profile in both C3 and Pz in A) with the CONTINUE beta profile in Figure 4A (right‐hand upper panels for Taps > 5).

First, we computed power differences and tested for significance via cluster‐based permutation tests (Fig. 5B). The data were subdivided into successful and unsuccessful stops. In order to select a principled cut‐off for this division, we iteratively varied the threshold of movement extent to define the outcome following the stop signal. Splitting the data with a range of movement extent thresholds from 36 to 48% consistently yielded significant clusters in both Pz and C3. Accordingly, we elected to use a movement threshold of 40% to discriminate successful and unsuccessful stops for further analyses (Figs 6A,B and 8C,D), in the knowledge that the results would be similar had we chosen a threshold from 36 to 48% of movement extent. The mean difference was strongest in Pz (Fig. 5B and C, *t*_16_ = 3.3, diff_avgPeak_ = 23.1% at 330 ms, 15 Hz, effect size = 1.1, *P *=* *0.004), whereas the highest *t*‐score was found in C3 (*t*_16_ = 4.1, diff_avgPeak_ = 21.2% at 375 ms, 14 Hz, effect size = 1.3, *P *=* *0.001), which was due to a larger between‐subjects variability in Pz (Fig. 6B).

Importantly, this power difference preceded the stop signal. Aligning low‐beta band time series data to the stop signal showed that the significant difference in C3 and Pz had in fact already ended about 100 ms before the stop signal (Fig. 6A). Furthermore, although differences in power occurred around the time of finger elevation between taps, power differences in Pz and C3 disappeared when trials were subdivided according to the median extent of finger upward movement (Fig. 6C). Subdividing the data according to the median tap‐to‐sound offset resulted in a significant difference that survived multiple‐comparison correction in C3 but not Pz and resembled the power difference related to stopping performance (Fig. 6D).

**Figure 7:**
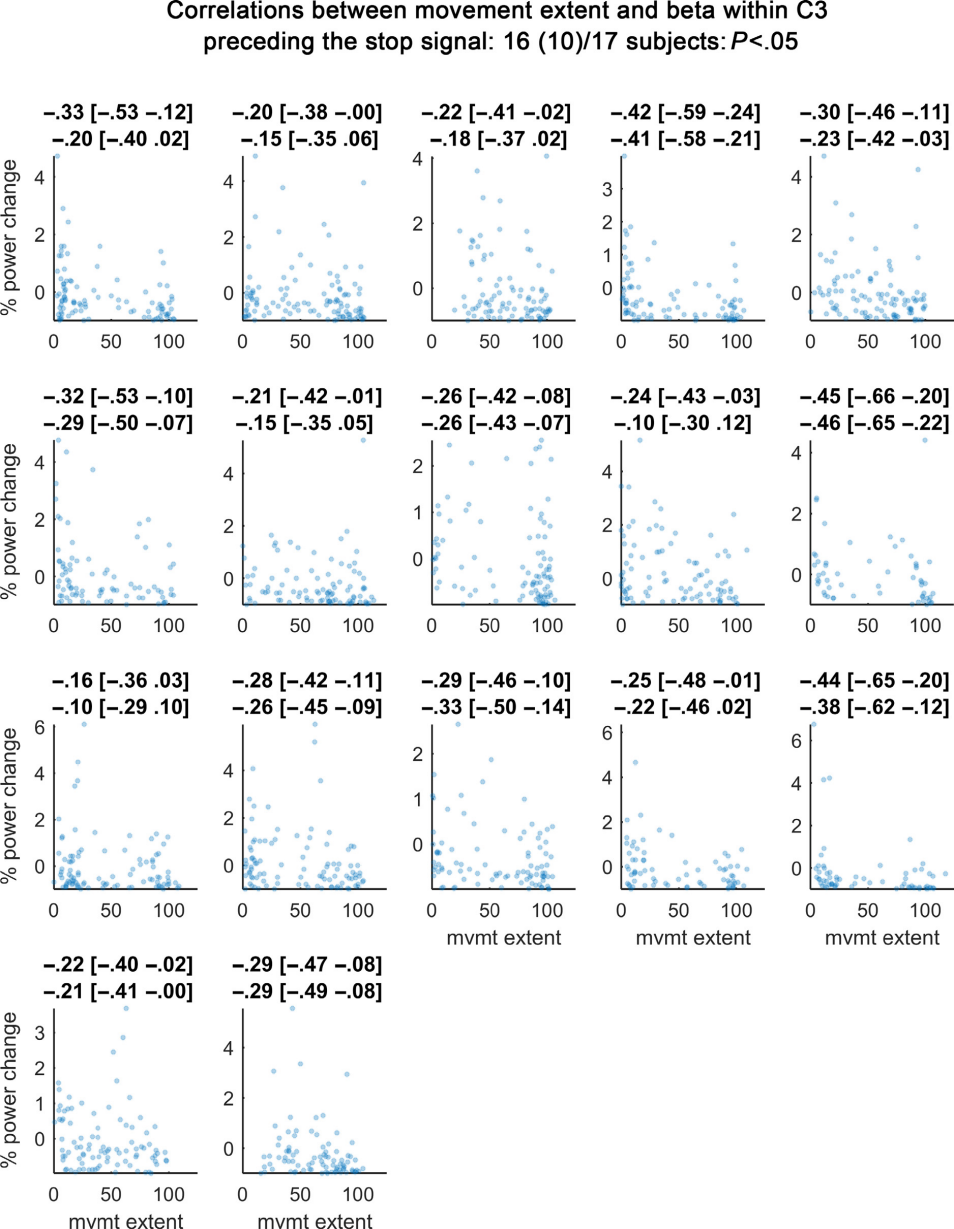
Scatter plot of correlations between movement extent (*x*‐axis) and beta relative to baseline (*y*‐axis). Subplots show individual participants. For each participant, beta power yielding the maximum correlation (detected anywhere between 12–30 Hz and 200–500 ms after the last regular tap considering that optimal frequencies and time points may differ across subjects) is shown. Plot titles denote Spearman's rho followed by its 95% bootstrapped confidence interval. The second line denotes the correlation coefficient resulting from the partial correlation controlling for the first two components obtained by principal component analysis of the behavioural variables. 16 of 17 subjects (10 if partial correlations were considered) had significant correlations.

In a next step, we investigated how many of the recorded subjects presented a significant correlation between stopping performance and power at any frequency in the beta band (12–30 Hz) in a 200–500 ms time window after the last regular tap. 16 of 17 participants showed a significant correlation between activity in the beta band in C3 and movement extent (Fig. 7). This was reduced to 10 of 17 participants after controlling for tapping parameters by performing partial correlations. Partial correlations were performed because several movement parameters also correlated with stopping performance (Table 2). To reduce the seven potentially related movement variables to two components accounting for most of the variance, we conducted a principal component analysis. The first two components accounted for on average 40 and 22% of the variance respectively. Scatter plots for Pz were similar to those from C3 (see Supporting Information Fig. S3), with 14 significant correlations (nine when computing partial correlations).

### EEG features that follow the stop signal and are linked to stopping performance

As a final question we asked if beta also was higher after the stop signal when the movement was inhibited.

**Figure 8:**
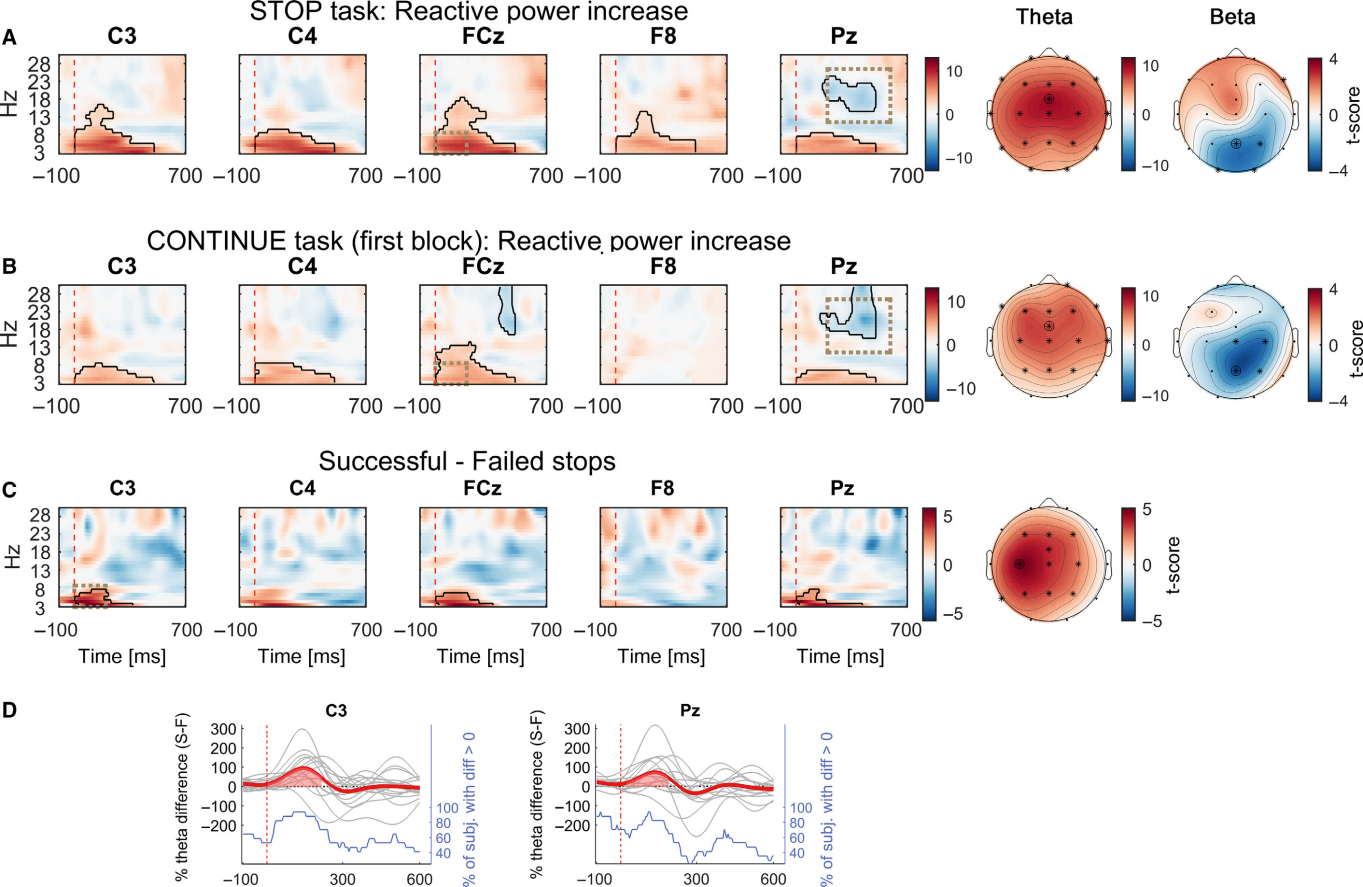
(A) *t*‐Scores of the contrast between power aligned to the stop signal (vertical dashed line) averaged across all STOP trials irrespective of stopping performance and the regular tap done before. (B) as (A) but computed on all CONTINUE trials of the first block instead. In clusters surrounded by black outlines, power differed significantly from power observed during the regular tap done before. Cluster‐based statistics were computed within 0 : 500 ms after the stop signal. Straight after the stop signal, power increased in low frequencies in all channels in both tasks. The cluster in the beta range denotes that beta power was lower relative to regular tapping. Topoplots show *t*‐scores from the average within the dashed rectangular windows: The low‐frequency power increase peaked fronto‐centrally, whereas the difference in beta was most pronounced over parietal and ipsilateral sites. (C) *t*‐Scores of power differences between more successful and less successful stops (movement extent threshold = 40%). Clusters denote that low‐frequency power was significantly higher when participants interrupted their tap before touching the pressure sensor. The corresponding topoplot shows that the *t*‐score was highest over contralateral M1. (D) Time courses of individual theta (3–8 Hz) power differences following the stop signal (vertical dashed line) corresponding to the difference depicted in (C). The bold line denotes the mean difference. The bottom stepped line shows for each point in time the fraction of participants who had higher theta power during successful stops.

Immediately after the stop signal, power increased significantly in low frequencies (Fig. 8A), as observed in classical stop signal reaction time tasks (Huster *et al*., 2013). In the STOP condition, the largest increase was observed in FCz (*t*_16_ = 8.3, diff_avgPeak_ = 428.3% at 240 ms, 3 Hz, effect size = 2.7, *P *<* *0.001). All but one subject displayed a significant power increase, thus this increase was highly consistent (Fig. 8D). Around 350 ms after the stop signal, parietal beta was lower than in the aftermath of the tap before (*t*_16_ = − 3.7, diff_avgPeak_ = − 19.9% at 365 ms, 19 Hz, effect size = − 1.1, *P *=* *0.002).

Both effects were also present in the first block of the CONTINUE condition (Fig. 8B) even though no immediate movement interruption was required or had been performed before. This power increase thus seemed to be linked to the processing of the salient stop signal rather than to actual motor inhibition. The reduced colour intensity in Fig. 8B compared to A suggests that the low‐frequency increase was smaller when tapping was continued than when it had to be stopped.

In a next step we compared whether power differed between successful and unsuccessful stops (Fig. 8C). When stopping was more successful, the low‐frequency power increase after the stop signal was significantly stronger than when it failed. The peak increase was strongest in FCz (*t*_16_ = − 4.9, diff_avgPeak_ = 166.5% at 225 ms, 3 Hz, effect size = 0.7, *P *<* *0.001), yet when the average within 0–200 ms and 3–8 Hz was computed, the topoplot showed clear lateralization with a stronger power increase over contralateral motor cortex (Fig. 8C). Correlations between movement extent and power within a 3–8 Hz and 0–200 ms time window after the stop signal were significant in 14 of 17 subjects (12 of 17 when computing partial correlations, see Supporting Information Fig. S4).

Taken together, only the low‐frequency, and not beta power following the stop signal, was associated with improved stopping ability. As it was higher in the STOP condition and during successful stops, this increase may have been modulated by the degree of attention to the signal, although we cannot directly test this hypothesis with the present data.

## Discussion

We found that movement‐related modulation in power at lower beta frequencies during rhythmic tapping increased with time on the task. This modulation was task‐dependent and less pronounced when sudden movement interruption was anticipated. In the latter case, tapping seemed to be more cautious as reflected by a reduced negative mean asynchrony and shorter contact duration between the finger and the pressure pad. More caution and less confidence in the continuity of the tapping task presumably implies increased cognitive load. However, when beta power modulation prior to the stop cue was more pronounced and more similar to the CONTINUE condition, it proved easier for participants to stop tapping. We hypothesize that increased confidence in tapping accurately on time was beneficial in terms of liberating resources that enable more efficient reaction to the unpredictable stop signal, and that this confidence and thus reduced cognitive load is reflected in more pronounced modulation of low‐beta power over parietal and motor cortical regions.

The posited association between confident movement execution and greater modulation of low‐beta power was supported by the temporal development of beta modulation. Modulation over contralateral motor cortex was visible after the first three taps in both tasks, but similar modulation over parietal cortex appeared to develop only in the power average displayed for the CONTINUE condition, consistent with more hesitant and less anticipatory tapping when an immediate reaction to a sudden stop signal was required.

The parietal cortex is thought to play a crucial role in maintaining an internal model during tapping to an isochronous metronome (Pollok *et al*., 2006, 2008). In line with this, reducing the excitability of the posterior parietal cortex via cathodal tDCS or rTMS reduces negative mean asynchrony (Krause *et al*., 2012, 2014). In addition, beta modulation in the form of increased post‐movement synchronization (albeit over sensorimotor cortex) has previously been associated with greater current confidence in the internal model of a task (Tan *et al*., 2014a,b; Tan *et al*., 2016). Similarly, a recent study has reported beta increase after successful stopping and a relative decrease following failed stops (Jha *et al*., 2015). These beta changes were found to be linked to the reaction time of the subsequent trial, such that a greater decrease in beta was associated with a larger increase in reaction time consistent with the notion of an error detection mechanism leading to reorganizational processes requiring cognitive resources.

We propose that within the STOP condition, confidence in how correctly taps were timed according to an internal model of the regular rhythm, fluctuated, and when it was higher, post‐movement beta power increased. This beta increase may have been linked to more efficient stopping by means of mediating enhanced connectivity between spatially disparate areas, which has been found to be high when attentional lapses are prevented (Gross *et al*., 2004). The relative decrease in parietal beta activity following the stop signal was also consistent with the above formulation. Attenuation of the post‐movement beta increase seemed to occur irrespective of whether the tapping movement was continued or not, presumably because the stop signal interfered with the internal representation of the sensorimotor synchronization model that was established to perform accurate tapping in both tasks.

How might our findings and interpretation be reconciled with the prevailing view that elevated beta power reinforces the current motor state, which in our paradigm was continued tapping (Engel & Fries, 2010)? This result could be reconciled if increased beta activity reinforced holding the finger in a lifted position between taps, delaying the reaction time of the next tap, which would be in line with previous findings, showing prolonged reaction times when beta was relatively high due to conflict, accuracy demands or uncertainty (Tzagarakis *et al*., 2010; Pastötter *et al*., 2012, 2013). However, if elevated beta activity was promoting stopping by maintaining the finger in its lifted position we would expect the power difference to be sustained rather than disappearing 100 ms prior to the stop signal. Moreover, this would not explain why the average modulation was reduced in the STOP condition. Rather we posit that decreases and increases in beta power infer a shift in neural activity away from and towards that promoting the status quo respectively (Jenkinson & Brown, 2011). Thus, beta reactivity may imply more flexible motor control, possibly through the facilitation of intra‐ and inter‐cortical communication (Rubino *et al*., 2006). Note, that enhanced pre‐stimulus parietal beta oscillations have previously also been linked to improved sensory detection and faster reaction times (Linkenkaer‐Hansen *et al*., 2004; Kaminski *et al*., 2012). In this context it has been proposed that increased beta may reflect a state of enhanced arousal rather than the status quo (Kaminski *et al*., 2012). However, against this interpretation is that we observed higher beta modulation in the undemanding CONTINUE condition, which was unlikely to induce greater arousal. Our results instead point towards a more complex involvement of beta oscillations in flexible motor control depending both on task demands and concurrent performance evaluation.

This study might be considered preliminary in that EEG sampling and hence spatial resolution was relatively limited. This, together with the relatively inferior signal‐to‐noise ratio of EEG, might explain why we could not confirm that successful stopping may be dependent on a beta increase in right inferior frontal cortex as reported in electrocorticography studies (Swann *et al*., 2009, 2012). In addition, it should be stressed that the correlations identified between beta power preceding the stop signal and stopping performance were relatively small, which might result from the noisy nature of EEG signals or genuinely weak interrelatedness. Another limitation of this study is the fact that the STOP and CONTINUE conditions were not counterbalanced and did not include matched numbers of trials. Contrasts between these conditions should therefore be treated with caution, although control analyses suggested that these confounds had relatively little effect.

In conclusion, our findings begin to link modulation of low‐beta oscillations to confidence in and reliance on an internally generated model of repetitive actions, which is continuously updated based on information received from the environment. In particular, pronounced oscillations in this frequency band may not only promote the current motor state or plan, as previously suggested (Gilbertson *et al*., 2005; Engel & Fries, 2010), but also appear to reflect reduced cognitive load, especially in conditions with additional task demands.

## Conflict of interest

The authors declare no conflict of interest.

## Supporting information

Fig. S1. Scatter plots of correlations between movement extent (*x*‐axis) and time spent in contact with the pressure sensor on the previous tap (downTime, *y*‐axis).
Fig. S2. Scatter plots of correlations between movement extent (*x*‐axis) and pressing vigour of the previous tap (maxPrs, *y*‐axis).
Fig. S3. Scatter plot of correlations between movement extent (*x*‐axis) and beta relative to baseline (*y*‐axis).
Fig. S4. Scatter plot of correlations between movement extent (*x*‐axis) and theta relative to baseline (*y*‐axis).
