# Supplementary material for: High post‐movement parietal low‐beta power during rhythmic tapping facilitates performance in a stop task
Source: Eur J Neurosci. 2016 Jul 20;44(5):2202–13. doi: 10.1111/ejn.13328 (PMC5014120; doi:10.1111/ejn.13328)
Supplement: Supplementary file 1 — Fig. S1. Scatter plots of correlations between movement extent (x‐axis) and time spent in contact with the pressure sensor on the previous tap (downTime, y‐axis). Fig. S2. Scatter plots of correlations between movement extent (x‐axis) and pressing vigour of the previous tap (maxPrs, y‐axis). Fig. S3. Scatter plot of correlations between movement extent (x‐axis) and beta relative to baseline (y‐axis). Fig. S4. Scatter plot of correlations between movement extent (x‐axis) and theta relative to baseline (y‐axis). [file EJN-44-2202-s001.pdf]

Supporting Information

1 Scatter plots of correlations between tapping parameters and movement extent

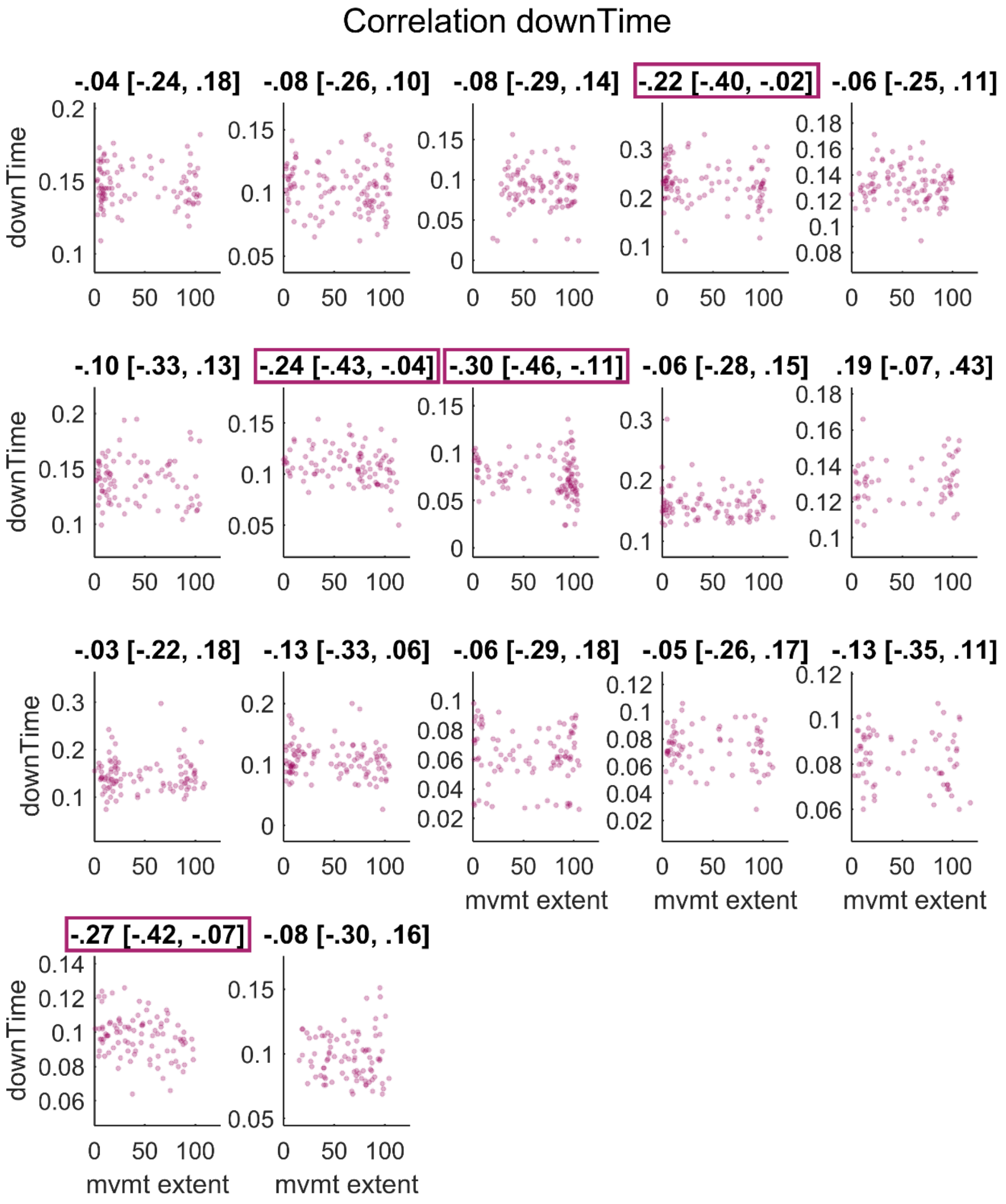

Supporting Fig. 1: Scatter plots of correlations between movement extent (x-axis) and time spent in contact with the pressure sensor on the previous tap (downTime, y-axis). Subplots show individual participants. Plot titles denote Spearman's rho followed by its 95% bootstrapped confidence interval. Only highlighted participants showed a significant correlation.

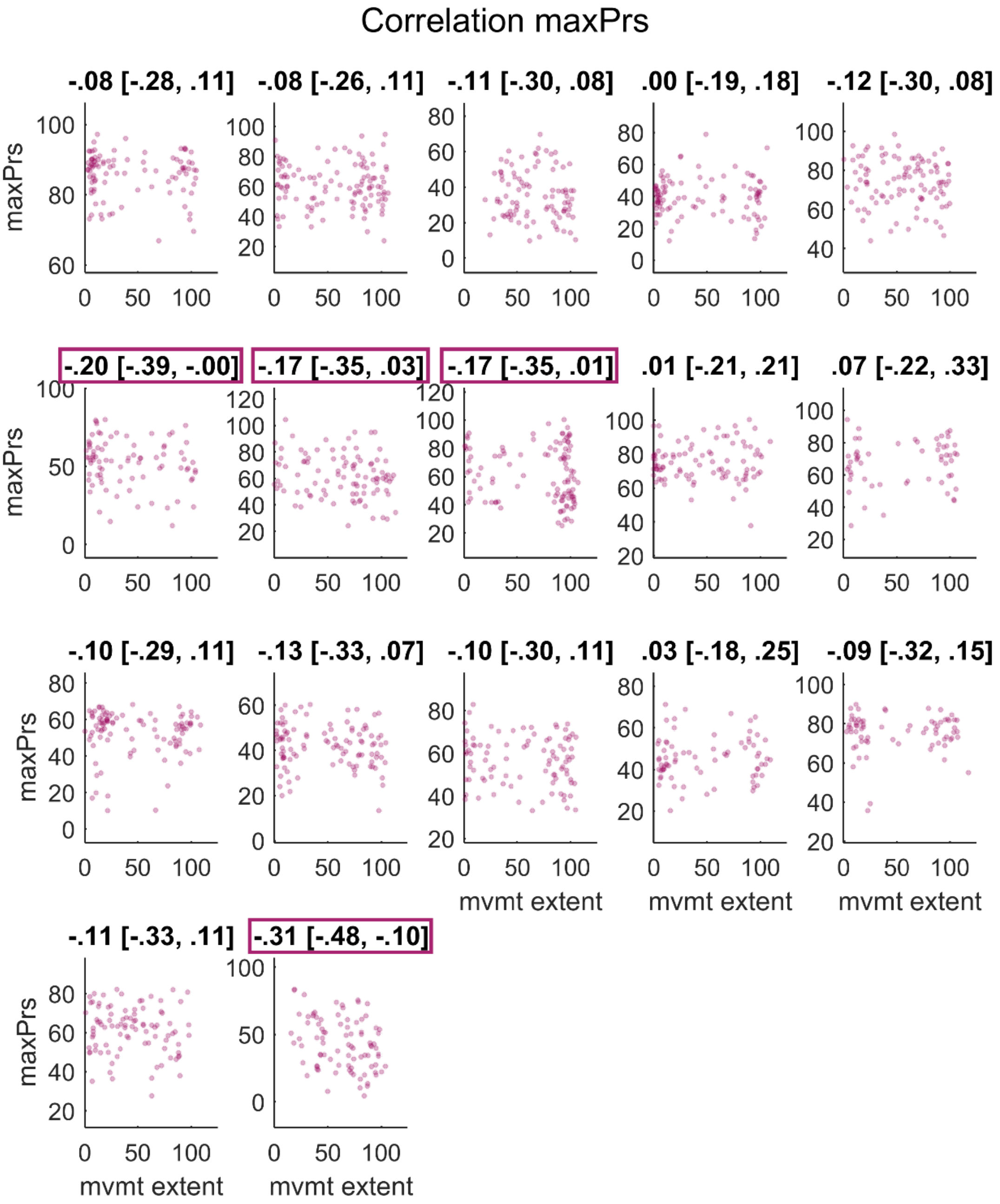

Supporting Fig. 2: Scatter plots of correlations between movement extent (x-axis) and pressing vigour of the previous tap (maxPrs, y-axis). Subplots show individual participants. Plot titles denote Spearman's rho followed by its 95% bootstrapped confidence interval. Only highlighted participants showed either a tendency to a correlation or significant correlation.

2 Control analyses for the STOP-CONTINUE condition comparison

To explore the validity of the comparison between the STOP and CONTINUE conditions we performed two additional control analyses. First, we examined if the behaviour and evolving beta reactivity differed between the two distinct blocks of the CONTINUE condition. Ideally they would not differ. Second, we matched the trials from the two conditions in number by selecting a reduced subset of trials from the middle of the STOP condition to make sure that condition differences did not result from differences in trial numbers.

2.1 Comparison of the first with the second CONTINUE block

2.1.1 Behaviour

We performed two 3x2 ANOVAs with tap-to-sound offsets and downTime as dependent variables and the two factors time (early, middle and late taps) and recording block (block 1 vs. 2).

The ANOVA with soundOffset as dependent variable resulted only in a significant main effect of time (Greenhouse-Geisser corrected $F_{2, 32} = 66.0$, $\varepsilon = .55$, $P < .001$) but no significant main effect of block ($F_{1, 16} = 0.3$, $\varepsilon = 1.0$, $P = .614$) or interaction (Greenhouse-Geisser corrected $F_{2, 32} = 2.7$, $\varepsilon = 0.67$, $P = .107$). The ANOVA with downTime as dependent variable showed no significant effects (time: Greenhouse-Geisser corrected $F_{2, 32} = 0.6$, $\varepsilon = .61$, $P = .491$; block: $F_{1, 16} = 0.2$, $\varepsilon = 1.0$, $P = .698$; time*block: Greenhouse-Geisser corrected $F_{2, 32} = 0.1$, $\varepsilon = .68$, $P = .800$). Hence blocks in the CONTINUE condition did not differ.

2.1.2 EEG

The ANOVA with low-beta modulation as dependent variable resulted again only in a significant main effect of time ($F_{2, 32} = 7.7$, $\varepsilon = .91$, $P = .002$, $mean_{early} = -0.9$, $mean_{middle} = 11.2$, $mean_{late} = 13.4$) but not block number ($F_{1, 16} = 0.4$, $\varepsilon = 1.0$, $P = .560$, $mean_{BLOCK1} = 7.1$, $mean_{BLOCK2} = 8.8$).

2.2 STOP-CONTINUE comparison with matched number of taps

For this comparison, trial numbers between the STOP and CONTINUE condition were matched by selecting a reduced subset of trials from the middle of the STOP condition discarding the same amount of data at the beginning and end of each participant's recording block. The two main effects found in the original ANOVA containing all trials were again significant (condition: $F_{1, 16} = 7.0$, $\varepsilon = 1.0$, P=.017, mean $_{CONTINUE}$ =7.6, mean $_{STOP}$ =0.4; time: $F_{2, 32} = 7.7$, $\varepsilon = .86$, $P = .003$, mean $_{early}$ = -2.6, mean $_{middle}$ = 7.3, mean $_{late}$ = 7.2).

3 Scatter plots of correlations between EEG power and movement extent

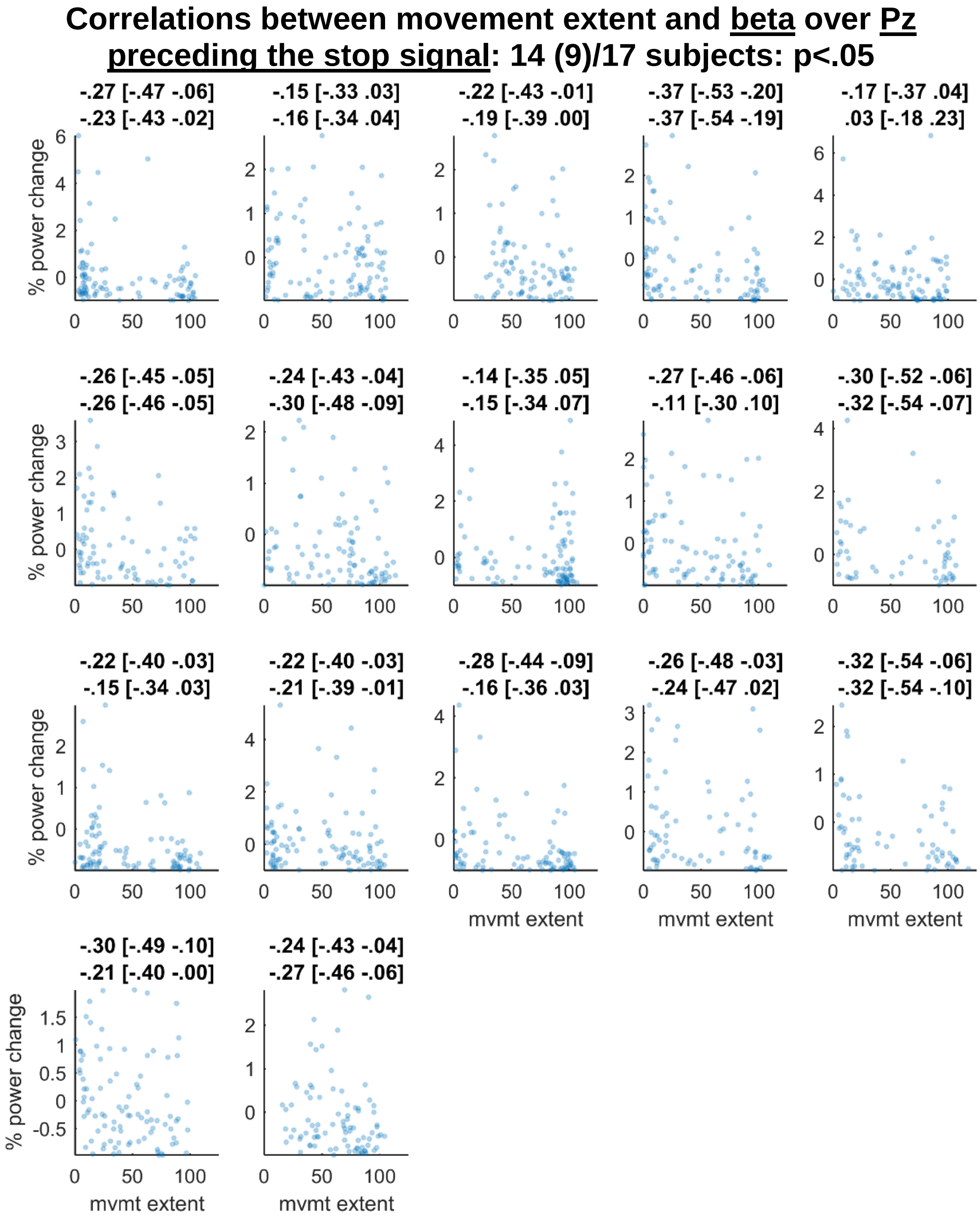

Supporting Fig. 3: Scatter plot of correlations between movement extent (x-axis) and beta relative to baseline (y-axis). Subplots show individual participants. For each subject, beta power yielding the maximum correlation (detected anywhere between 12-30 Hz and 200-500ms after the last regular tap considering that optimal frequencies and time points may differ across subjects) is shown. Plot titles denote Spearman's rho followed by its 95% bootstrapped confidence interval. The line below denotes the correlation coefficient resulting from the partial correlation controlling for the first two components obtained by principal component analysis of the behavioural variables. 14 of 17 subjects (9 if partial correlations were considered) had significant correlations.

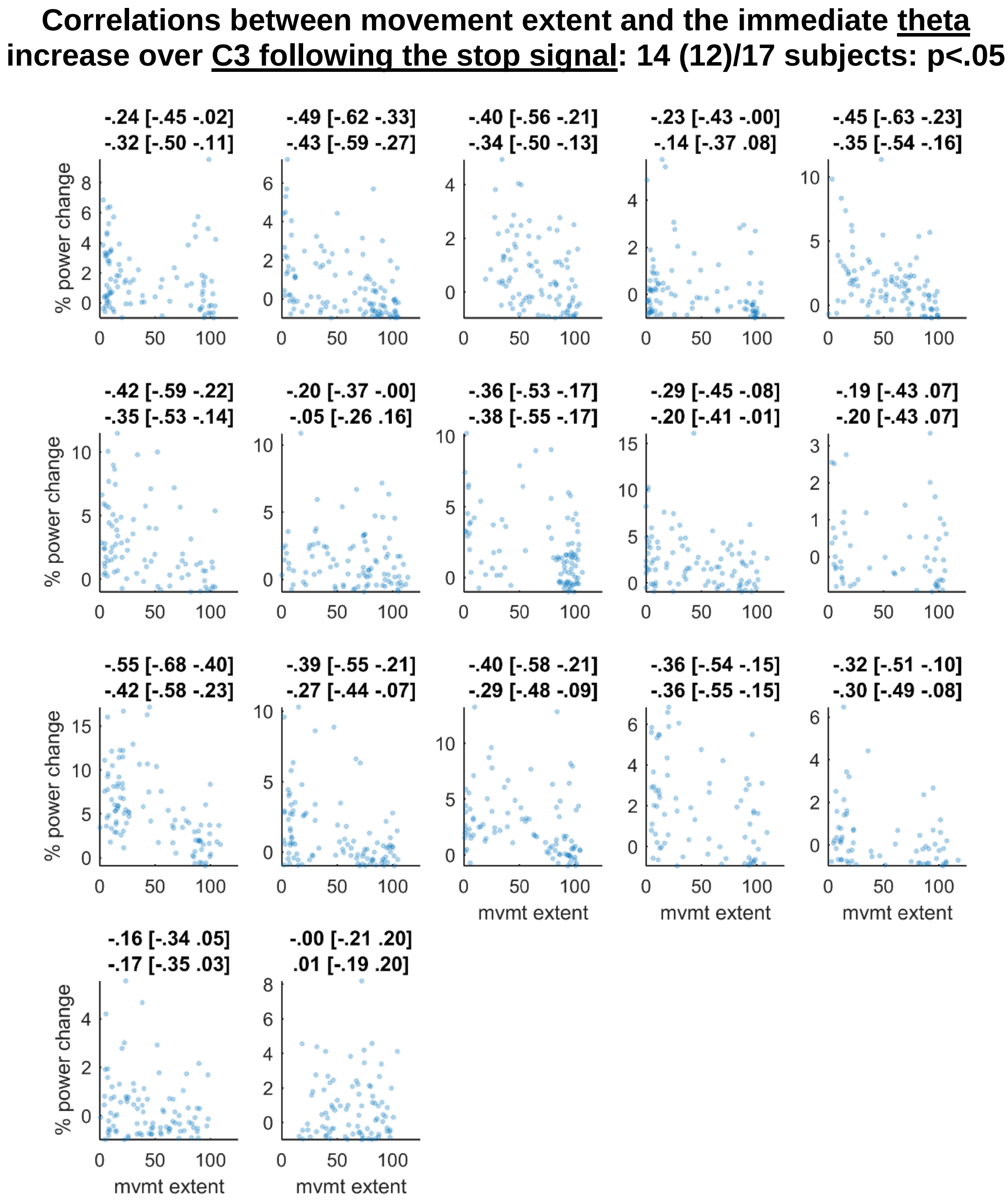

Supporting Fig. 4: Scatter plot of correlations between movement extent (x-axis) and theta relative to baseline (y-axis). See figure legend of Supporting Fig. 3**.** The maximum correlation was detected anywhere between 3-8 Hz and 0-200ms after the stop signal.
